# Unintended Childbearing and Child Growth in Northern Malawi

**DOI:** 10.1007/s10995-016-2124-8

**Published:** 2016-08-04

**Authors:** Angela Baschieri, Kazuyo Machiyama, Sian Floyd, Albert Dube, Anna Molesworth, Menard Chihana, Judith R. Glynn, Amelia C. Crampin, Neil French, John Cleland

**Affiliations:** 1 Faculty of Epidemiology and Population Health, London School of Hygiene and Tropical Medicine, London, UK; 2 Community Health Department, College of Medicine, University of Malawi, Zomba, Malawi; 3 School of Clinical Sciences, University of Edinburgh, Edinburgh, UK; 4 Karonga Prevention Study, Karonga, Malawi; 5 Institute of Infection and Global Health, University of Liverpool, Liverpool, UK

**Keywords:** Fertility intentions, Fertility preference, Fertility, Child nutrition, Child growth, Stunting, Unintended pregnancy, Propensity Score Matching, Malawi, Africa

## Abstract

*Objective* The study aims to assess whether unintended children experience slower growth than intended children. *Methods* We analysed longitudinal data linked to the Karonga Health and Demographic Surveillance Site collected over three rounds between 2008 and 2011 on women’s fertility intentions and anthropometric data of children. Using the prospective information on fertility intention we assessed whether unintended children are more likely to be stunted than intended children. We applied Propensity Score Matching technique to control for endogenous factors affecting both the probability that a family has an unwanted birth and a child with poor health outcomes. *Results* We found that 24 % of children from unwanted pregnancies were stunted compared with 18 % of mistimed pregnancies and 17 % of those from wanted pregnancies. However, these differences in probability of children being stunted, though in the expected direction, were not significant either for large or small families, after controlling for age. The number of children in the household was associated with stunting and boys were substantially more likely to be stunted than girls. *Conclusion* We found no significance difference in probability of being stunted by mother’s fertility intention.

## Significance

Studies that assess health consequences of unintended childbearing using retrospective data on fertility intentions such as the Demographic and Health Surveys suffer from *ex post rationalization* which is the propensity to report children wanted when they were originally unwanted. In addition, in cross-sectional studies it is problematic to establish a causal link between unintended childbearing and child health. This is the first prospective study in Africa to assess the health consequences of unintended childbearing on child growth, we analyse longitudinal data on children growth and data on fertility intentions collected before the birth of the child.

## Introduction

Although there is wide agreement that the promotion of family planning lowers fertility, it is not clear to what extent greater contraceptive use and smaller family sizes will enhance health and family welfare or what are the consequences of unintended childbearing on children’s life chances (Lloyd 1994; Lloyd and Montgomery 1996; Montgomery and Lloyd 1997; Rosenzweig 1990; Schultz 2005; National Research Council 1986). Unintended births include those births occurring whilst parents are desiring to delay pregnancy and births to parents who wish to have no more children.

In this paper, the effects of unintended childbearing on child growth are examined. Under nutrition is the underlying cause of 35 % of the burden of disease in under-five children; low weight-for-age, low-height-for age and low-weight-for-height are all associated with increased risks of death (Black et al. 2008). Longer term consequences, including reduced stature in adulthood, cognitive impairment and chronic disease, are also apparent (Hoddinott et al. 2013).

These adverse health effects also have economic implications. The accumulation of human capital is thought to be one of the key elements of sustainable economic growth (Barro 1990; Becker 1965; Becker et al. 1990). The importance of an educated workforce for economic growth has been widely documented and demonstrated (Denison 1962; Denison and Chung 1976; Psacharopoulos 1981). The role of health has received much less attention, but convincing evidence exists of the importance of investment in public health for positive economic performance (Strauss 1986; Fogel 1994). Early childhood health influences the achievement of traits that are rewarded in the labour market such as improved cognitive performance, higher educational attainment, and positive personality attributes (Hoddinott et al. 2013; Palloni 2006).

In sub-Saharan Africa, the lack of attention to this issue has been detrimental to the development of sound economic policy. Reproductive health issues are routinely overlooked by the Poverty Reduction Strategy Papers supported by the World Bank and other international donors. Furthermore, only in 2007 was “universal access to reproductive health” added to the Millennium Development Goals. A lack of investment in future generations is one of the factors keeping African economies in a ‘poverty trap’.

We inform this debate by analyzing data collected within an on-going Demographic Surveillance Site (DSS) in Karonga District in Northern Malawi. Malawi is one of the poorest countries in the world; it has high fertility, with a Total Fertility Rate (TFR), according to the 2010 Demographic and Health Survey, of 5.7 births per woman, with 42 % of married women using any method of contraception. It is estimated that 26 % of women have an unmet need for contraception (National Statistical Office (NSO) and ICF Macro 2011). Furthermore, close to half of women with six or more children said that ideally they would have liked fewer than six children (National Statistical Office (NSO) and ICF Macro 2011). Around 47 % of children under 5 years old in Malawi are moderately stunted, or too short for their age, and 20 % severely stunted (National Statistical Office (NSO) and ICF Macro 2011). The DSS of Karonga district provides the ideal setting to study the relationship between family planning and fertility and investment in children’s nutrition as it generates detailed longitudinal data on a range of demographic, nutritional and socio-economic characteristics of both households and individuals. The key question to be addressed is whether the health and growth of children whose conception was unintended is adversely affected either because of conscious or unconscious discrimination in terms of nutrition or health care or because the advent of an unwelcome child threatens overall household welfare and in particular food security.

This is the first prospective study in Africa to assess the health consequences of unintended childbearing on child growth. Married or cohabiting couples were asked whether they wanted more children and the preferred timing of future births. Over the 3 year follow-up period, new births could thus be classified as intended (wanted), or unintended (unwanted or mistimed). Using these data on prospective fertility intentions, which were reported before the conception leading to the birth of the children, we assessed whether unintended recently born children are more likely to be stunted than intended children and if these differences are greater for children living in large families than in small families. Stunting was chosen as the key outcome, because it can be measured objectively and because it is the best overall indicator of longer-term health and nutrition.

## Methods

This study uses data collected between October 2008 and October 2011 from a module on fertility intentions linked to the on-going Karonga DSS in Northern Malawi, and repeated anthropometric data of children aged 10 years or younger collected in three rounds in 2008–2009, 2009–2010 and 2010–2011. The DSS baseline census was conducted in 2002-2004 following which the population has been under continuous surveillance. Details of the demographic data collection procedures have been documented (John et al. 2007; Crampin et al. 2012).

To assess the effect of unintended childbearing on child growth a set of questions to measure prospective fertility intentions of couples was designed in July and August 2008, translated into the local language (Chitumbuka) and piloted from September to October 2008. Data collection began on October 28th 2008.

Women and men of reproductive age were asked questions on their current fertility and their marital status. A section on fertility preferences was introduced in order to assess separately whether or not the husband and wife wish to have another child and the preferred timing of the next birth. Together with anthropometric data from each survey round, we are able to analyse the effect of being unintended on the growth of a newly born child.

As some mothers were either pregnant or did not respond to all the three rounds of the fertility intention study, we use the first available statement on preferences to define subsequent births as wanted, mistimed or unwanted. Unwanted births were defined as births occurring to women who wanted no more children. Mistimed births are those occurring within 6 months to women who wished to wait 1–2 years, those occurring within 18 months to women who wished to wait 2–3 years and those occurring within 30 months to those wishing to wait 3 or more years. The intendedness of the children with missing information on the mother’s fertility intention and those born before the mothers’ first report on fertility intention were classified as ‘unknown’. All other births were defined as intended. In this study, husbands’ preferences are not analyzed; this is justified by the high level of agreement (80 %) between spouses on desire for another child which has already been described (Baschieri et al. 2013). We restricted our analyses to children aged 1 year or older because of low stunting during infancy due to the protective effect of breastfeeding. A preliminary analysis showed that less than 7 % of children under 1 year old were stunted in our sample compared to more than 20 % amongst the children 1 or 2 years old.

We calculated the probability of being stunted at the last available examination for children born during the 3 years follow-up using the 2000 US CDC Growth Reference curve. Height-for-age Z scores were calculated with the STATA command zanthro and stunting is defined as Z-score of less than -2 (Vidmar et al. 2004).

Between 2007 and 2008 a household questionnaire on household dwelling characteristics and possessions was administered to all the households in the study site. We use this information to measure the household’s welfare status. We linked this information to all the children in our sample. In addition, an individual socio-economic survey was administered every year containing information on source of income, education and food security for all persons living in the DSS area. We linked this information on the mothers to all their children in our sample using the socio-economic survey data prior to the time when their first statement of fertility intentions was obtained. Similarly, household sero-surveys collected HIV status for all residents of the area. We link the mother’s sero-status prior to when their fertility intention was first asked.

We used bivariate analysis to analyse these relationships. In order to assess the impact of mothers’ childbearing intentions on the nutritional status of the children we applied Propensity Score Matching (PSM) analysis. This method controls for the fact that ‘exposed’ children (exposed to one of the events described above, or being a result of ‘unwanted’ or ‘mistimed’ births) are also more likely to be from lower socio-economic groups; hence in order to establish the ‘true’ effect of exposure on child health outcomes, it is necessary to control for this confounding. PSM creates a propensity score for each child in the ‘exposed’ group (given a set of background characteristics) and then matches the child to a control, those not exposed to the event with a similar score. We then can compare the outcome (i.e. stunting) for the ‘exposed’ and ‘unexposed’ children.

To achieve this matching, the PSM technique uses a logistic regression based on a set of background characteristics to calculate the probability or propensity of each respondent of being exposed (Rosenbaum and Rubin 1983). After assigning the propensity score to each individual, those with similar propensity scores are matched using alternative matching methods such as radius matching and kernel matching. After assessing the quality of the matching we estimate the Average Exposure Effect which is the difference in the outcome of interest amongst the exposed group and a similar group of children who were not exposed (Rosenbaum and Rubin 1983). In order to assess whether the effect is significant we estimated bootstrapped standard errors around the estimates (Rosenbaum and Rubin 1983; Lechner 2002).

Ethical approval for the study was received from the National Health Sciences Research Committee, Malawi (NHSRC Protocol No. 520), and the ethics committee of the London School of Hygiene & Tropical Medicine, UK. Before the start of the demographic surveillance the Traditional Authority that covers the area, and all village headmen and traditional advisors in the study area were informed about the aims of the study and the nature of the data to be collected, and their approval and verbal authorisation was sought. All household members were given a similar explanation and interviews were only conducted if verbal consent was given by the household head and by the respective household members. The socio-demographic data for this study come from the basic demographic surveillance for which the ethics committees agreed that written consent was not needed. For the fertility intentions survey and for HIV testing, separate individual written consent was sought from the individuals with information sheets in the local language, Chitumbuka.

## Results

Among 8768 women under 50 years old who participated in the fertility intentions survey, 3978 mothers had at least one child during the 3 year period of the study (28 October 2008–13 October 2011). Of this sample, 12 % of children did not have anthropometric data or had a biologically implausible value of height for age. We excluded from the analysis children who were under 1 year of age because the stunting level are low during this period and their growth pattern is influenced by breastfeeding. We analysed the remainder of 1704 children to 1701 mothers in this study (Table 1).

**Table 1:** Distribution of children by intendedness

	N (Children)
Known	
Wanted	190
Mistimed	160
Unwanted	125
Total number of children	475
Unknown	
No prospective fertility intention data	23
Child born before the first fertility intention statement	1206
Total number of children	1229
All children	1704

Among 2639 children who born between 28 October 2008 and 13 October 2010, we excluded children who (a) died in infancy (79 children), (b) were examined before the first birthday (620 children), (c) were not examined at all (206 children) or (d) had biologically implausible values in height for age (30 children)

The probability of being stunted was significantly higher for children in households with 4 or more children (25 vs 19 %), children of HIV positive mothers (38 vs 21 %), and children of mothers who experienced a prior child death (29 vs 19 %). The prevalence of stunting declined monotonically with increasing maternal education. Stunting also depended on household income with children living in poor and agricultural households being more likely to be stunted than richer households or households with a salary employment as their main source of income (Table 2).

**Table 2:** Percentage stunted among children aged 1–2 years, by mother’s and household characteristics

	Number of children aged 1–2		Stunted (%)	*p* value
	%	N		
Mother’s age				
<30 years old	72.7	1238	19.6	*
30 years or older	27.3	466	25.3	
Number of children in household				
1–3 living children	63.3	1158	19.4	**
4+ living children	37.7	546	24.9	
Mother’s HIV status				***
Positive	4.6	1468	38.5	
Negative	86.1	78	21.0	
Missing	9.3	158	14.6	
Experience of child death				**
Yes	17.5	299	29.1	
No	68.2	1162	19.4	
Missing	14.3	243	20.2	
Mother’s education				*
None/Primary 1–5	13.8	235	26.4	
Primary 6–8	59.6	1016	21.7	
Secondary 1–3	19.6	333	18.3	
Secondary 4/Higher	5.2	89	10.1	
Don’t know	1.8	31	20.8	
Marital status				
Not married	13.3	226	21.7	
In monogamous union/unknown	77.8	1325	20.6	
In polygamous union	7.6	130	26.1	
Missing	1.3	23	21.7	
Asset index				**
Poorest	20.6	351	24.2	
Poor	20.0	341	23.2	
Middle	21.1	360	23.1	
Rich	18.7	319	19.7	
Richest	13.1	223	11.2	
Missing	6.5	110	23.6	
Household size				
1–4 people	39.9	690	20.9	
5–6 people	27.1	532	18.4	
7 or more	31.6	458	24.9	
Missing	1.4	24	20.8	
Main source of income: farming				**
Yes	74.2	1264	23.0	
No	23.0	393	15.5	
Missing	2.8	47	19.2	
Salary employed				*
Yes	10.0	170	13.5	
No	86.4	1472	22.1	
Missing	3.6	62	21.00	

* *p* < 0.05; ** *p* < 0.01; *** *p* < 0.001

We found that 24 % of children from unwanted births were stunted compared with 18 % of those mistimed and 17 % of those who were wanted and 19 % for those whose wantedness status was not measured (Table 3). However, the differences between children from wanted, mistimed and wanted births, though in the expected direction, were not significant either for large or small families. The probability of being stunted in children born since the beginning of the fertility intentions study increased with age. Boys were more likely to be stunted than girls (26 vs 17 %).

**Table 3:** Percentage stunted among children aged 1–2 years old by intendedness and child’s characteristics

	All Children			In smaller families (1–3 living children)			In larger families (4+ living children)		
	Stunting (%)	N		Stunting (%)	N		Stunting (%)	N	
All children born after the start of the study	21.2	1704		20.1	1048		22.9	656	
Unknown intendedness	22.1	1229		21.5	783		23.1	446	
Known intendedness	18.9	475		16.2	265		22.4	210	
All children born with intended status									
Wanted	16.8	190		14.3	140		24.0	50	
Mistimed	17.5	160		18.0	89		16.9	71	
Unwanted	24.0	125		19.4	36		25.8	89	
Gender			*	***		**			**
Male	25.6	864	*	24.2	537		27.8	327	
Female	16.7	840	*	15.8	511		17.9	329	
Age in years									
1	20.1	1125		19.8	706		20.5	419	
2	23.3	579		20.8	342		27.0	237	

* *p* < 0.05; ** *p* < 0.01; *** *p* < 0.001

We repeated the analysis controlling for age, gender, and number of living children (Table 4). Predicted probabilities of stunting for children from wanted, mistimed and unwanted births were 18.8, 18.0 and 24.6 %, respectively. Though the differences were in the expected direction they were not statistically significant, either among small or large families. We also assessed whether mortality differed by intendedness among all new children. During the study period, 149 children died. The percentages who died among unwanted and mistimed children were higher than among wanted children (3.0, 3.1 and 2.3 %) but the differences were not significant.

**Table 4:** Predicted probabilities of being stunted by intendedness, adjusted for age, gender and total number of living children

Intendedness	Predicted probability	95 % CI	
		Lower	Higher
Wanted	18.76	13.37	25.68
Mistimed	17.97	12.47	25.21
Unwanted	24.64	17.35	33.74
Unknown	20.94	18.55	23.52
In smaller families (1–3 living children)			
Wanted	14.24	9.13	21.53
Mistimed	16.99	10.37	26.57
Unwanted	18.63	8.90	34.97
Unknown	21.13	18.27	24.33
In larger families (4 or more living children)			
Wanted	32.23	18.56	47.50
Mistimed	20.62	11.84	33.43
Unwanted	31.48	21.32	43.78
Unknown	19.74	13.56	24.29

The PSM model matched individuals on a range of demographic and socio-economic characteristics which were selected considering the association with the probability of being stunted and exposure. We matched the exposed and unexposed samples on child’s age, the gender of the child, the mother’s education, mother’s marital status, whether or not she experienced the death of a child, her HIV status, the total number of children in the household, household head’s occupational status, household welfare, whether or not the household had a phone and whether they have enough food in the household.

Table 5 shows the results of the PSM procedure. The proportion of children stunted amongst those children from unwanted births (first row, first column) can be compared to the proportion of children stunted among the matched sample of non- exposed individuals, those from wanted or mistimed births (first row, second column). The second row shows the Average Exposure Effect (AEE). Row 3 and 4 show the bootstrap standard error and its significance level (the matching procedure is bootstrapped to obtain the true standard errors around the simulated AEE).

**Table 5:** Results of Propensity Score Matching analysis for the effect of fertility intentions amongst children aged 1–2 years

Rows		All children	
		Unwanted	Wanted
1	Percentage stunted	24.00	20.97
2	Average exposure effect (AEE)	3.02	
3	Bootstrap SE	0.035	
4	*P* value	<0.447	
5	Obs	1704	

The propensity score results can be considered valid if the matching removes the difference in underlying measured characteristics between the exposed and unexposed samples. The mean absolute variance before and after matching is shown in row 8 and 9 and the number of variables with absolute bias of more than 10 % before and after matching.

We found a 3 % points difference between the probabilities of being stunted for those who were unwanted and those wanted or mistimed. The model performed well and we managed to remove all the difference between the two groups (children wanted and children unwanted or mistimed) in the matched sample. However, when we simulated the standard errors of these estimates with bootstrap and we found that these difference was not statistically significant.

## Discussion

In the United States a large, complex and inconclusive literature exists on the possible disadvantages that children whose conception was unintended may have in comparison to children who were intended (Brown and Eisenberg 1995). In developing countries, it is estimated that about 40 % of pregnancies are either unwanted or mistimed and, of these, half are terminated, 13 % end in miscarriages and 38 % are taken to term (Singh et al. 2010). In view of this high incidence of unintended births, together with a high prevalence of poverty, it is surprising how few relevant studies have investigated whether children resulting from unintended pregnancies have lower survival and poorer health outcomes than children whose conceptions were intended. Montgomery et al. used Demographic and Health Survey data from five countries to assess the effects of unwantedness on child survival, nutrition and education (Montgomery and Lloyd 1997). Unwantedness was weakly linked to mortality in three countries and to nutrition in one country. Marston and Cleland also used Demographic and Health Survey data from five countries to assess whether pre- and post-natal health care and outcomes differed between children classified by mothers as arising from wanted, unwanted and mistimed births (Marston and Cleland 2002). Only in one country did they find a link between unwantedness and childhood stunting. In India, an analysis using a mother-level fixed effects model found a negative association between unintendedness and survival (Singh et al. 2012). These studies rely on retrospective reports by the mother of intendedness and are thus subject to the criticism that mothers’ recall of pregnancy intention may be influenced by the subsequent survival or growth of the child. To our knowledge, only three studies, two in India and one in Bangladesh, have used a longitudinal design to assess the link between fertility intentions reported in advance of conception and subsequent survival of children (Singh et al. 2013; Chalasani and Casterline 2007; Bishai et al. 2015). Two found higher neonatal mortality among unintended than intended births while the third found no effect on mortality. This impression of scarcity of evidence and mixed results is confirmed by a review of the literature (Gipson et al. 2008).

This study was conducted in a poor rural setting where under-nutrition is common. Unlike most previous studies, it has the advantage of prospective fertility preference information, thus circumventing the problem of *post*-*facto* rationalisation encountered when retrospective preference data are used. Nevertheless, no statistically significant difference was found in the nutritional status according to whether the child’s conception was wanted at that time, was mistimed or not wanted at all, according to the mother’s reported preference stated before conception. Differences were in the expected direction and it is possible that a larger sample size would have established a significant effect but one of modest magnitude. It is reasonable to conclude that unintended children suffer no subconscious or conscious discrimination in terms of food allocation or health care. It is more surprising that the stress of the advent of an unintended birth on household income and food security in this poor and under-nourished population was not reflected in the growth of the index child; however this effect might appear in older siblings.

A possible explanation for this negative result is that questions on fertility intentions are poorly understood by women in Karonga or that responses fluctuate over time. In a society where contraception is absent the former hypothesis would be convincing but, in Karonga, 43 % of married women reported use of a method and thus the idea of volitional childbearing and reproductive control is not alien. The fact that a high level of agreement was found between spouses concerning desire for another child also indicates that fertility intention data are valid. We were also able to check consistency of women’s intentions over the course of the study. Among women who said at baseline that they wanted no more children, 35 % were pregnant at round 3 or had given birth, 13 % had changed their intention to wanting another child or were unsure while 53 % gave the same response (Machiyama et al. 2015). The salient feature of reproduction in Karonga appears to be weak implementation of fertility intentions rather than volatility of intentions, a verdict supported by the fact that contraceptive use in this setting is subject to high discontinuation and incorrect use (Das Gupta et al. 2015).

Other interpretations are more plausible. As throughout sub-Saharan Africa, traditions in Karonga no doubt place a high value on children and positive emotional reactions to a new birth may outweigh any prior intentions to avoid or delay further births. Moreover, unwanted and mistimed births are commonplace in this population. One of the remarkable findings of the study is that over one-third of young children were born to mothers who had earlier stated a desire for no more and a similar proportion were mistimed. In a setting where fertility control is weak and unintended childbearing is so common, it is perhaps unsurprising that no adverse effect of unintendedness was found relative to other children.

The analysis found that boys are more likely to be stunted than girls, confirming similar results from other studies (Griffiths et al. 2004). However, elucidation of the reason may require ethnographic or biological study.

What are the policy implications? Malawi has a national family planning programme whose ultimate purpose is to reduce unwanted childbearing. The apparent absence of any link between unintended births and nutritional welfare found in this study in no way undermines investment in family planning. Greater and more effective use of contraception will inevitably lead to a reduction of unintended pregnancies and to smaller family sizes, where there is less competition between siblings for scarce resources. Improvements in family welfare are likely to result from this transition.
